# A Modified Vancomycin Molecule Confers Potent Inhibitory Efficacy against Resistant Bacteria Mediated by Metallo-β-Lactamases

**DOI:** 10.3390/molecules27227685

**Published:** 2022-11-09

**Authors:** Le Zhai, Ya Liu, Yue Jiang, Ling-Yan Kong, Jian Xiao, Yi-Xue Wang, Yang Shi, Yi-Lin Zhang, Ke-Wu Yang

**Affiliations:** 1 Shaanxi Key Laboratory of Phytochemistry, Engineering Research Center of Advanced Ferroelectric Functional Materials, College of Chemistry and Chemical Engineering, Baoji University of Arts and Sciences, Baoji 721013, China; 2 Key Laboratory of Synthetic and Natural Functional Molecule of the Ministry of Education, College of Chemistry and Materials Science, College of Life Sciences, Northwest University, Xi’an 710127, China; 3 College of Biology Pharmacy and Food Engineering, Shangluo University, Shangluo 726000, China

**Keywords:** antibiotic resistance, metallo-β-lactamases, vancomycin, triazolylthioacetamide, β-lactam antibiotics

## Abstract

Multidrug-resistant bacterial infections mediated by metallo-β-lactamases (MβLs) have grown into an emergent health threat, and development of novel antimicrobials is an ideal strategy to combat the infections. Herein, a novel vancomycin derivative **V_b_** was constructed by conjugation of triazolylthioacetamide and vancomycin molecules, characterized by reverse-phase high performance liquid chromatography (HPLC) and confirmed by matrix-assisted laser desorption/ionization-time of flight mass spectrometry (MALDI-TOF MS). The biological assays revealed that **V_b_** effectively inhibited *S. aureus* and methicillin-resistant *S. aureus* (MRSA), gradually increased the antimicrobial effect of β-lactam antibiotics (cefazolin, meropenem and penicillin G) and exhibited a dose-dependent synergistic antibacterial effect against eight resistant strains tested, which was confirmed by the time-kill curves determination. Most importantly, **V_b_** increased the antimicrobial effect of meropenem against the clinical isolates EC08 and EC10 and *E. coli* producing ImiS and CcrA, resulting in a 4- and 8-fold reduction in MIC values, respectively, at a dose up to 32 μg/mL. This work offers a promising scaffold for the development of MβLs inhibitors, specifically antimicrobials for clinically drug-resistant isolates.

## 1. Introduction

Bacterial resistance has become a global problem threatening human life and health [1]. Multidrug-resistant bacterial infections are on the rise as many clinical antibiotics have been rendered ineffective [2]. Among them, resistant Gram-positive *Enterococcus faecium* and *Staphylococcus aureus* are listed as high priorities for new treatments in “ESKAPE pathogens” and in “the list of drug-resistant bacteria” released by the World Health Organization recently [3,4]. Gram-negative pathogens, such as *P. aeruginosa* and *K. pneumoniae*, have presented an enormous clinical challenge due to the dearth of effective antibiotics against these bacteria [5]. In addition, superbugs mediated by metallo-β-lactamases (MβLs) are almost resistant to clinically used antibiotics, including penicillins, cephalosporins and carbapenems [6,7].

Vancomycin (Van), a clinically glycopeptide antibiotic, is known as the “antibiotic last resort” for the treatment of Gram-positive bacterial infections, especially for methicillin-resistant *S. aureus* (MRSA) [8,9]. However, over the past decades, the increased clinical use of vancomycin has led to the emergence of vancomycin-resistant bacteria, including vancomycin-resistant *S. aureus* (VRSA) and *E. faecium* (VRE), which has become a new challenge for antibacterial therapy [10,11,12]. Vancomycin specifically binds to the D-Ala-D-Ala terminal of the cell-wall pentapeptide precursor to inhibit the cell wall biosynthesis of Gram-positive bacteria [13]. Bacteria acquired resistance to vancomycin by mutating the pathogen peptidoglycan sequence from D-Ala-D-Ala to D-Ala-D-Lac, resulting in an overall 1000-fold decrease in the binding affinity to vancomycin [14,15].

At present, VRE has become one of the most common acquired pathogens in hospitals and the treatment options for these drug-resistant infections are severely limited, which has created an urgent need for new clinical agents with activity against resistant pathogens [16]. Thus, various modification strategies of novel vancomycin derivatives have been developed to combat vancomycin resistance, such as lipophilic modification of vancomycin, enhancing the binding affinity of the drug for bacterial ligands, pyrophosphate-targeting designs of cell wall phospholipids and the modification of the vancomycin main structure by total synthesis [17,18,19,20,21]. Recently, our group creatively modified the photosensitizer porphyrin onto the vancomycin molecule; the photosensitizer porphyrin–vancomycin molecule was targeted and enriched on drug-resistant bacteria cells and then the Gram-positive bacteria were inactivated at a specific wavelength by photodynamic therapy [22]. Furthermore, it has been reported that the lipophilic and cationic motifs on vancomycin were modified in combination to enhance the ability of vancomycin to penetrate the bacterial membrane, including vancomycin derivatives carrying C-terminal lipophilic quaternary ammonium moieties and carrying the lysine-rich lipopeptides [23,24]. It was found that vancomycin derivatives with hydrophobic substituents in the disaccharide moiety have significant antibacterial activity against drug-resistant strains including MRSA and VRE [25]. Recently, Venkateswarlu et al. developed a dipicolyl–vancomycin (Dipi-van) conjugate as an inhibitor for the NDM-1 enzyme, which has the ability to penetrate the outer membrane of Gram-negative pathogens (GNPs) and reinstate the activity of carbapenem [26].

**Scheme 1:**
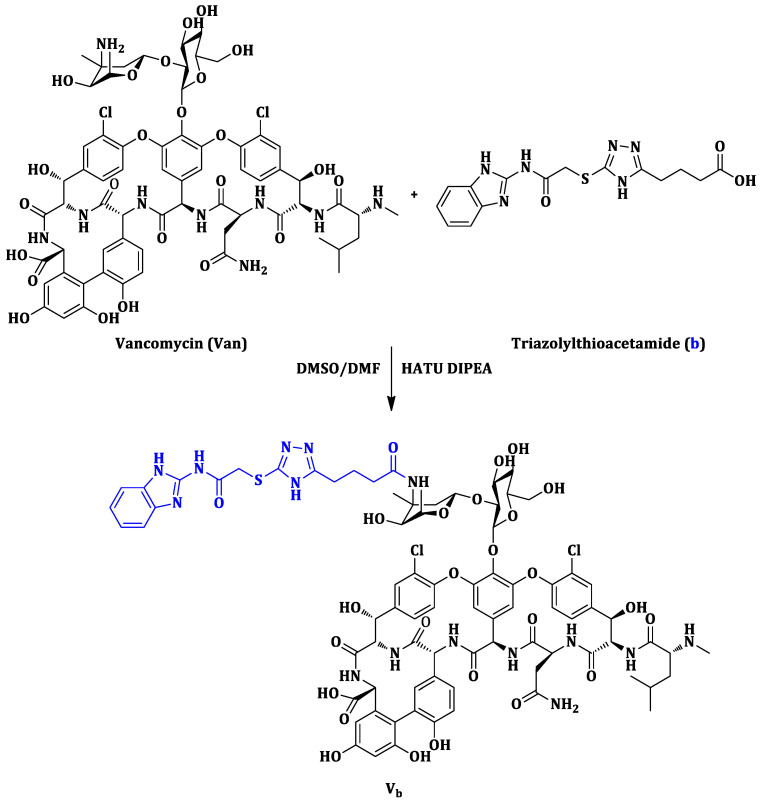
Synthetic route of the vancomycin derivative **V_b_**.

We have previously reported the triazolylthioacetamides with different substitutional groups, which exhibited good inhibitory activity against the bacterial resistance target MβLs and restored the antibacterial activity of antibiotics against *P. aeruginosa* and ImiS-producing *E. coli* [27,28]. In this work, we constructed a novel vancomycin derivative **V_b_** (Scheme 1) by modifying the molecule with a hydrophobic triazolylthioacetamide that has low steric hindrance and a carboxyl group (see Supplementary Materials). **V_b_** was characterized by reverse-phase HPLC and confirmed by MALDI-TOF MS. The antibacterial activity of **V_b_** and its antibacterial activity synergizing with β-lactam antibiotics against resistant Gram-negative bacteria that produce MβLs and Gram-positive bacteria were evaluated (see Supplementary Materials).

## 2. Results and Discussion

The synthetic pathway of the vancomycin derivative **V_b_** is shown in Scheme 1. Triazolylthioacetamide (**b**) was synthesized with previously reported methods [27], characterized by ^1^H and ^13^C NMR, and further confirmed by MS (see Supplementary Materials). The synthesis of the vancomycin derivative **V_b_** was adapted from literature procedure [13,22]. Briefly, vancomycin hydrochloride and the synthesized triazolylthioacetamide **b** were dissolved in 2 mL dry cosolvent (DMF/DMSO = 1/1) at 0 °C. Then a solution of HATU in DMF was added dropwise, followed by diisopropylethylamine (DIPEA). The reaction mixture was allowed to stir for 20 h at room temperature. The resulting crude product was loaded onto a Sephadex G-25 column to offer the purified **V_b_** as a white powder with a total yield of 18%.

**Figure 1:**
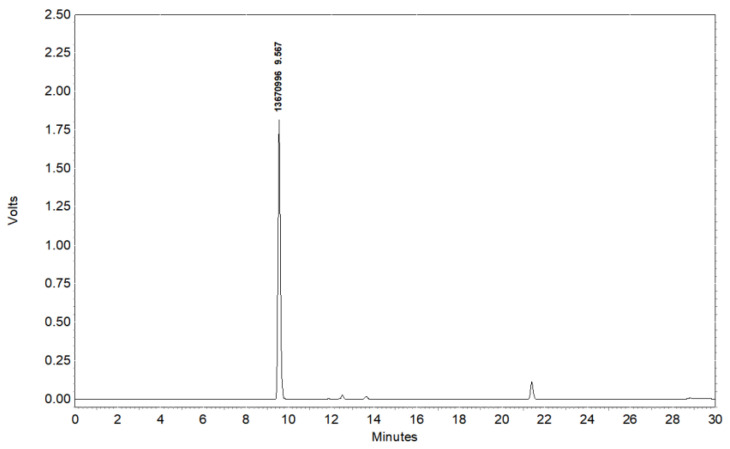
HPLC analysis of vancomycin derivative **V_b_**.

The obtained **V_b_** was analyzed by reverse-phase HPLC using a C18 column (4.6 × 250 mm) and a UV detector (280 nm), and the column was eluted with a gradient of 5–70% acetonitrile containing 0.1% TFA in 30 min at a flow rate of 1 mL/min. The liquid product **V_b_** was first treated with a 0.22 μm filter membrane. The HPLC analysis result for **V_b_** is shown in Figure 1, indicating that the purity of this compound was more than 95%.

**Figure 2:**
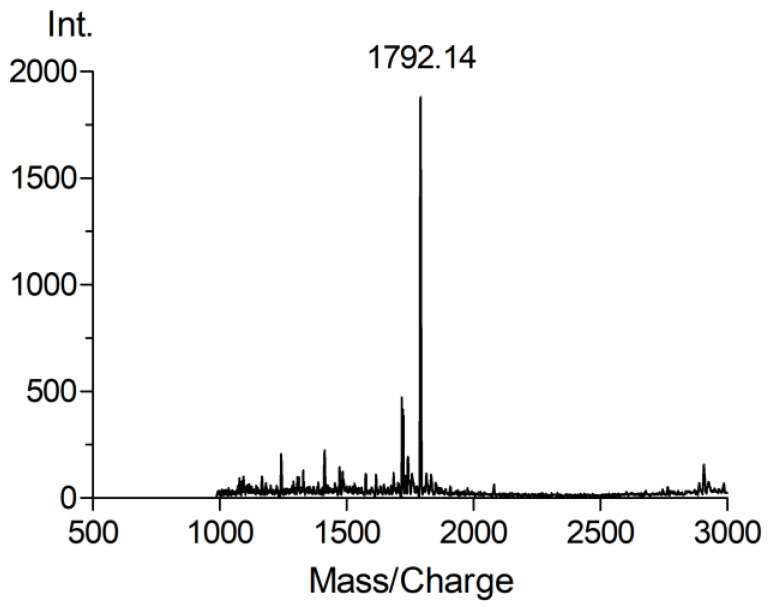
MALDI-TOF mass spectrum of vancomycin derivative **V_b_**.

The purified **V_b_** (white powder) was confirmed by MALDI-TOF MS. As shown in Figure 2, the peak at 1792.14 (*m/z*: calculated for [M+H]^+^ = 1792.63) corresponding to **V_b_** was clearly observed, demonstrating that triazolylthioacetamide **b** was conjugated with the vancomycin successfully.

The antibacterial activities of vancomycin and **V_b_** were evaluated in vitro by determining the minimum inhibitory concentrations (MICs) according to the Clinical and Laboratory Standards Institute (CLSI) broth micro-dilution method [29]. The employed resistant Gram-positive pathogens were *S. aureus*, MRSA and VRE. Resistant Gram-negative bacteria were *K. pneumoniae*, the clinical isolates *E. coli* producing New Delhi metallo-β-lactamases (NDMs), including *E. coli* 08 (EC08), *E. coli* 10 (EC10) and *E. coli* BL21 (DE3) producing MβL ImiS or MβL CcrA. The collected MIC data are summarized in Table 1.

The collected MIC data indicated that **V_b_** had effective antibacterial activity against *S. aureus* and MRSA, which was similar to the parent vancomycin molecule. However, the low antimicrobial activities against Gram-negative bacteria and MβL-producing resistant strains were also observed. Next, we assessed the synergistic effects of **V_b_** with three β-lactam antibiotics (cefazolin, meropenem and penicillin G) against the above eight resistant strains. The MIC values for Gram-positive *S. aureus*, MRSA, VRE and Gram-negative *K. pneumoniae* are listed in Table 2, and for four MβLs-producing bacteria are listed in Table 3.

The MIC data indicated that **V_b_** gradually increased the antimicrobial effect of all tested β-lactams with an increasing dose and exhibited a dose-dependent synergistic antibacterial effect against the eight resistant strains. The highest dose of **V_b_** (4 μg/mL) resulted in a maximum 128-fold MIC decrease in the antibiotics against *S. aureus* and MRSA, respectively, and a dose of 16 μg/mL **V_b_** resulted in a 128-fold MIC decrease in cefazolin against VRE. **V_b_** also increased the antimicrobial effect of all tested β-lactams against *K. pneumoniae*, resulting in a 16–32-fold reduction in MICs. Importantly, as shown in Table 3, **V_b_** increased the antimicrobial effect of meropenem against the clinical isolates *E. coli* 08 and *E. coli* 10, resulting in a 4-fold reduction in MIC value at a dose up to 32 μg/mL. Furthermore, **V_b_** resulted in an 8-fold MIC decrease in the antibiotics against resistant *E. coli* producing ImiS and CcrA at a dose of 32 μg/mL.

**Figure 3:**
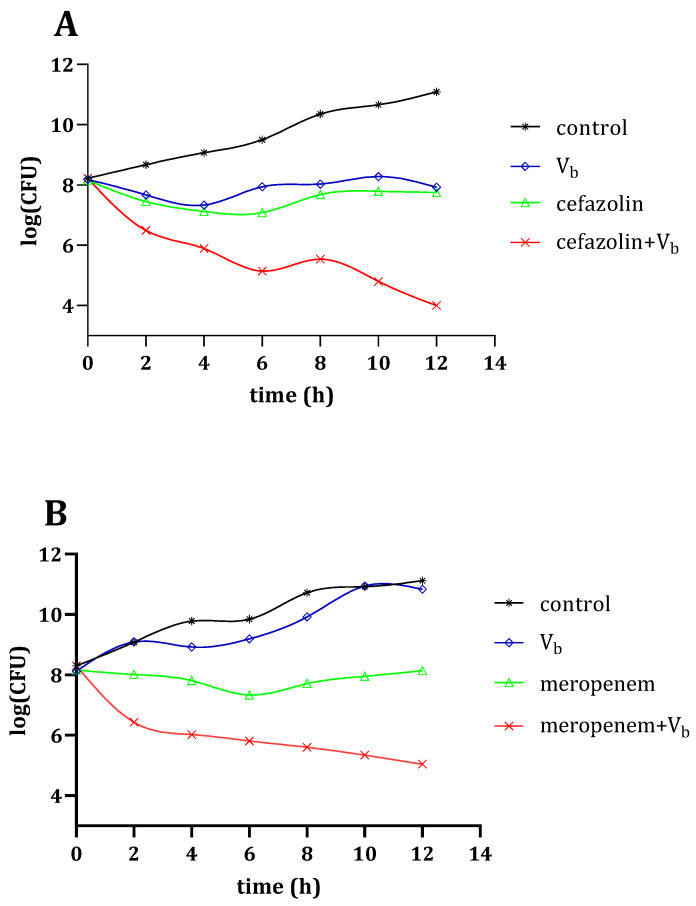
Time-kill kinetic analysis of **V_b_**-, antibiotics- and synergetic therapy-treated *S. aureus* (**A**) and *K. pneumonia* (**B**) for 12 h.

The potent synergistic antibacterial activity of **V_b_** was verified by time-kill curves (see Supplementary Materials) against *S. aureus* and *K. pneumonia* as shown in Figure 3. Figure 3A shows that the population of *S. aureus* decreased after treatment with **V_b_** alone for 12 h, indicating that **V_b_** has bactericidal activity against *S. aureus*. Furthermore, compared with cefazolin treatment alone, the synergistic therapy of cefazolin with **V_b_** resulted in a significant reduction in the population of *S. aureus*.

Furthermore, Figure 3B shows that the population of *K. pneumonia* at the exponential phase is significantly reduced upon exposure to the synergistic therapy of meropenem with **V_b_** for 12 h. Indeed, the time-kill curves against *S. aureus* and *K. pneumonia* confirmed the synergistic antibacterial effect of **V_b_** with β-lactam antibiotics.

## 3. Conclusions

A novel vancomycin derivative **V_b_** was constructed by conjugation of the triazolylthioacetamide **b** and vancomycin molecule, characterized by reverse-phase HPLC and confirmed by MALDI-TOF MS. The biological assays showed that **V_b_** had effective antibacterial activity against *S. aureus* and MRSA, but low antimicrobial activities against Gram-negative *K. pneumoniae*. Moreover, **V_b_** gradually increased the antimicrobial effect of three β-lactam antibiotics tested (cefazolin, meropenem and penicillin G), exhibited a dose-dependent synergistic antibacterial effect against the eight resistant strains, and the synergistic effect of **V_b_** and β-lactam antibiotics against *S. aureus* and *K. pneumonia* was confirmed by the time-kill curves determination. Most importantly, **V_b_** increased the antimicrobial effect of meropenem against the clinical isolates EC08 and EC10 and *E. coli* producing ImiS and CcrA, resulting in a 4- and 8-fold reduction in MIC value at a dose up to 32 μg/mL. This work offers a promising scaffold for the development of MβLs inhibitors, specifically antimicrobials for clinically drug-resistant isolates.

## Figures and Tables

**Table 1:** Antibacterial activities (MICs, μg/mL) of vancomycin and vancomycin derivative **V_b_** against the resistant Gram-positive and Gram-negative strain at the diluted concentrations from 4 to 512 μg/mL.

Strains	Vancomycin (μg/mL)	V_b_ (μg/mL)
*S. aureus*(ATCC29213)	4	4
MRSA(ATCC43300)	4	8
VRE	512	512
*K. pneumoniae*	>512	>512
EC08	512	>512
EC10	512	>512
*E. coli* (producing ImiS)	512	>512
*E. coli* (producing CcrA)	512	>512

**Table 2:** Antibacterial activities (MICs, μg/mL) of vancomycin derivative **V_b_** synergizing with β-lactam antibiotics against the resistant strains at a dose in the range of 1–32 μg/mL.

*S. aureus* (ATCC29213)				
	Control ^a^	+1 ^b^	+2 ^b^	+4 ^b^
Cefazolin	0.25	0.25	0.125	0.0156
Meropenem	0.03125	0.03125	0.0156	0.00048
Penicillin G	0.5	0.3125	0.156	0.0039
MRSA (ATCC43300)				
	Control ^a^	+1 ^b^	+2 ^b^	+4 ^b^
Cefazolin	4	4	0.5	0.03125
Meropenem	0.5	0.5	0.25	0.0039
Penicillin G	8	8	4	0.0625
VRE				
	Control ^a^	+4 ^b^	+8 ^b^	+16 ^b^
Cefazolin	2	0.5	0.125	0.0156
Meropenem	1	0.25	0.0625	0.0156
Penicillin G	32	16	2	1
*K. pneumoniae*				
	Control ^a^	+8 ^b^	+16 ^b^	+32 ^b^
Cefazolin	1250	625	156	78
Meropenem	0.125	0.0625	0.03125	0.0078
Penicillin G	1250	312.5	156	39

^a^ Control: MIC values of antibiotics alone against the tested bacterial strain; +1 ^b^ to +32 ^b^: MIC values of antibiotics against the tested bacterial strain in the presence of 1 to 32 μg/mL **V_b_**.

**Table 3:** Antibacterial activities (MICs, μg/mL) of vancomycin derivative **V_b_** synergizing with β-lactam antibiotics against resistant *E. coli* producing MβLs (NDMs, ImiS and CcrA) at a dose of 8, 16, and 32 μg/mL.

EC08 (Producing NDMs)				
	Control ^a^	+8 ^b^	+16 ^b^	+32 ^b^
Cefazolin	5000	5000	5000	2500
Meropenem	128	64	32	32
Penicillin G	>20,000	>20,000	>20,000	20,000
EC10 (producing NDMs)				
	Control ^a^	+8 ^b^	+16 ^b^	+32 ^b^
Cefazolin	2500	2500	2500	1250
Meropenem	64	64	32	16
Penicillin G	10,000	10,000	10,000	5000
*E. coli* (producing ImiS)				
	Control ^a^	+8 ^b^	+16 ^b^	+32 ^b^
Meropenem	64	32	16	8
*E. coli* (producing CcrA)				
	Control ^a^	+8 ^b^	+16 ^b^	+32 ^b^
Cefazolin	32	32	32	4

^a^ Control: MIC values of antibiotics alone against the tested bacterial strains; +8 ^b^ to +32 ^b^: MIC values of antibiotics against the tested bacterial strains in the presence of 8 to 32 μg/mL **V_b_**.

## Data Availability

Not applicable.

## Supplementary Materials

The following supporting information can be downloaded at: https://www.mdpi.com/article/10.3390/molecules27227685/s1, Materials and instruments; Synthesis of vancomycin derivative **V_b_**; Antibacterial activity assay in vitro; Time-kill kinetic analysis. All four Supplementary Materials references have been appeared in the maintext’s reference list: the first ref is in line with Ref. [27]; second ref is in line with Ref. [13]; third ref is in line with Ref. [22]; The last is in line with Ref. [29].
