# Supplementary material for: A Modified Vancomycin Molecule Confers Potent Inhibitory Efficacy against Resistant Bacteria Mediated by Metallo-β-Lactamases
Source: Molecules. 2022 Nov 9;27(22):7685. doi: 10.3390/molecules27227685 (PMC9693118; doi:10.3390/molecules27227685)
Supplement: Supplementary file 1 [file molecules-27-07685-s001.zip › molecules-1952096-supplementary.pdf]

Supplementary Material

A modified vancomycin molecule confers potent inhibitory efficacy against resistant bacteria mediated by metallo-β-lactamases

Le Zhai [1,*], Ya Liu [2,†], Yue Jiang [1,†], Ling-Yan Kong [2,†], Jian Xiao [1], Yi-Xue Wang [1], Yang Shi [1], Yi-Lin Zhang [3] and Ke-Wu Yang [2,*]

1 Shaanxi Key Laboratory of Phytochemistry, Engineering Research Center of Advanced Ferroelectric Functional Materials, College of Chemistry and Chemical Engineering, Baoji University of Arts and Sciences, Baoji 721013, China

2 Key Laboratory of Synthetic and Natural Functional Molecule of the Ministry of Education, College of Chemistry and Materials Science, College of Life Sciences, Northwest University, Xi'an 710127, China

3 College of Biology Pharmacy and Food Engineering, Shangluo University, Shangluo 726000, China

* Correspondence: zhaile@bjwlxy.edu.cn (L.Z.); kwyang@nwu.edu.cn (K.-W.Y.)

† These authors contributed equally to this work.

Content

1. Materials and instruments······3

2.Synthesis of vancomycin derivative $\mathbf{V}_b$······3

3. Antibacterial activity assay *in vitro*······4

4. Time-kill kinetic analysis······4

5. References ······5

1. Materials and instruments

General chemicals were purchased from Sigma Aldrich and were used without further

purification. *S. aureus* (ATCC29213), MRSA (ATCC43300), VRE, *K. Pneumoniae*, MβLs-producing clinical strains *E. coli* 08, 10 (EC08, EC10) were obtained from the Health Science Center of Xi'an Jiaotong University and the Fourth Military Medical University, China. Luria-Bertani (LB) medium and Mueller-Hinton (MH) broth were used as the growth media for bacterial strains and were obtained from OXOID. ^{1}H and ^{13}C NMR spectra were recorded on a Bruker Avance III 400 MHz NMR spectrometer. UV-Vis absorbance spectra were recorded on a UV 8454 spectrometer (Agilent, U.S.A.). The reverse-phase HPLC analysis was performed on LC-10 (Shimadzu, Japan) instrument with a C-18 column (4.6 × 250 mm) and UV detector (280 nm). MS spectra were measured with an AXIMA-CFR™ plus MALDI-TOF-mass spectrometer (Kratos Analytical, U.K.).

2. Synthesis of vancomycin derivative V_b

The triazolylthioacetamide **b** was synthesized as a white powder with a yield of 62% with previously reported method[1]. The synthesized compound was characterized by ^{1}H NMR and confirmed by HRMS. The data is as follows:

^{1}H NMR (400 MHz, DMSO-d_6): δ 12.06 (s, 1H), δ 7.88 (d, 2H), 7.40 (d, 2H), 4.20 (s, 2H), 2.70 (t, 2H), 2.56 (t, 2H), 1.87 (m, 2H). HRMS (ESI) *m/z*: obsd. 359.0999 (Calcd for $[M-H^{+}]^{-}$: 359.0921).

The synthesis of vancomycin derivative $\mathbf{V_b}$ (Figure 1) was adapted from a literature procedure[2, 3]. Vancomycin hydrochloride (150 mg, 0.10 mmol, 1 equiv) was dissolved in 2 mL dry cosolvent (DMF/DMSO = 1/1). The synthesized triazolylthioacetamide **b** (72 mg, 0.20 mmol, 2 equiv) was added and the mixture was cooled to 0°C. Then a solution of HATU (0.15 mmol, 1.5 equiv) in DMF was added dropwise, followed by DIPEA (0.5 mmol, 5 equiv). The reaction mixture was allowed to warm to room temperature and stirred for 20 h. Then the crude product was loaded onto a Sephadex G-25 column and eluted with ultrapure water at a flow rate of 1.0 mL/min. Eluent was collected every 2 mL per tube and monitored by UV-Vis spectrometer.

3. Antibacterial activity assay *in vitro*

The MIC values were determined by the Clinical and Laboratory Standards Institute

(CLSI) broth micro-dilution method[4]. The antibacterial activity of the vancomycin derivative **V_b** was investigated by determining the MIC values in sterile 96-well plates. The bacteria tested include *S. aureus* (ATCC29213), MRSA (ATCC43300), VRE, *K. pneumonia*, MβLs-producing clinical strains *E. coli* 08, 10 (EC08, EC10) and *E. coli* BL21 (DE3) containing plasmids pET26b-CcrA, pET26b-ImiS. Briefly, a single colony of the bacteria on agar plates was transferred into 10 mL of the Mueller-Hinton (MH) culture medium. Strains grown in the MH medium to OD_{600} of 0.5 were used as inoculum after an 84-fold dilution to 1×10^5 CFU/mL in the MH medium. The antibiotics and **V_b** was dissolved in the MH medium to prepare solutions of different concentrations required for the experiment. The as-prepared **V_b** solutions with different concentrations (50 μL) and MH medium/antibiotics solutions with different concentrations (50 μL) were added to a 96-hole plate, and then 100 μL inoculum was added sequentially to the solutions. The MH medium (200 μL) containing bacteria without anything were used as control. MIC was interpreted as the lowest concentration of the drug that completely inhibited the visible growth of bacteria after incubating plates at 37 °C for at least 16 h. Each measurement was performed in triplicate in at least two independent experiments and the highest MIC value was reported.

4. Time-kill kinetic analysis

For time-kill kinetic experiments, single colony of *S. aureus* and *K. pneumonia* on LB agar plates was transferred into 10 mL of MH liquid medium and grown at 37 °C overnight. The bacterial cells were collected by centrifugation (10000 rpm, 5 min). After discarding the supernatant, the pelleted cells were re-suspended in MH medium and diluted to 1×10^8 CFU/mL. *S. aureus* was treated with the final concentration of 1 μg/mL cefazolin, 8 μg/mL **V_b** and synergetic therapy, respectively. *K. pneumonia* was treated with 0.5 μg/mL meropenem, 16 μg/mL **V_b** and synergetic therapy, respectively. And the MH medium containing bacteria without anything were used as blank control. The mixture was incubated at 37 °C for 12 h, and the numbers of colonies (CFU/mL) were enumerated on LB agar every two hours. And 500 μL of this bacterial suspension was mixed with 500 μL of MH containing twice the desired final concentrations of **V_b**, antibiotics and synergetic therapy, respectively.

5. References

[1]. Yang SK, Kang JS, Oelschlaeger P, Yang KW. Azolylthioacetamide: A Highly Promising Scaffold for the Development of Metallo-β-lactamase Inhibitors. ACS Medicinal Chemistry Letters. 2015; 6(4): 455–460.

[2]. Yarlagadda V, Sarkar P, Samaddar S, Haldar J. A Vancomycin Derivative with a Pyrophosphate-Binding Group: A Strategy to Combat Vancomycin-Resistant Bacteria. Angewandte Chemie International Edition. 2016; 55(27): 7836–7840.

[3]. Zhai L, Yang KW. Porphyrin-vancomycin: A highly promising conjugate for the identification and photodynamic inactivation of antibiotic resistant Gram-positive pathogens. Dyes and Pigments. 2015; 120: 228–238.

[4]. CLSI, Methods for dilution antimicrobial susceptibility tests for bacteria that grow aerobically. 11th ed. CLSI standard M07, Wayne, PA: Clinical and Laboratory Standard Institute; 2018.
